# Sparing Muscle While Losing Fat: A Narrative Review of Ketogenic Diets and Lean Mass Preservation During Weight Loss

**DOI:** 10.3390/nu18172933

**Published:** 2026-09-07

**Authors:** Alfredo Caturano, Edoardo Mocini, Luca Titi, Francesco Leva, Daniele Cannavaro, Maria Grazia Tarsitano, Caterina Conte

**Affiliations:** 1 Department of Human Sciences and Promotion of the Quality of Life, San Raffaele Roma University, 00166 Rome, Italy; alfredo.caturano@uniroma5.it (A.C.); francesco.leva@uniroma5.it (F.L.); caterina.conte@uniroma5.it (C.C.); 2 Department of Theoretical and Applied Sciences, eCampus University, 22060 Novedrate, Italy; edoardo.mocini@uniecampus.it; 3 Department of Emergency-Admissions, Critical Care and Trauma, Policlinico Umberto I University Hospital, Associated with Sapienza University of Rome, 00161 Rome, Italy; luca.titi@policlinicoumberto1.it; 4 Obesity and Obesity-Related CardioMetabolic Complications Research Unit, IRCCS MultiMedica, 20099 Milan, Italy; daniele.cannavaro@multimedica.it

**Keywords:** ketogenic diet, very-low-energy ketogenic diet, lean mass, fat-free mass, skeletal muscle, body composition, weight loss, obesity, muscle protein synthesis, resistance exercise

## Abstract

**Background/Objectives:** Clinically meaningful weight loss reduces fat mass but is commonly accompanied by loss of fat-free mass (FFM). Ketogenic diets are of interest because ketone bodies have plausible anabolic and anti-catabolic actions. This narrative review examined whether these mechanisms translate into preservation of lean compartments or skeletal muscle during weight loss in clinical studies. **Methods:** MEDLINE was searched via PubMed from database inception to 14 August 2026 using terms related to ketogenic exposure, body composition, muscle metabolism, and weight loss. Evidence was synthesised according to dietary context, energy and protein intake, population, exercise exposure, measurement modality, and timing relative to carbohydrate restriction or reintroduction. **Results:** Acute human studies demonstrate biological effects of ketone bodies on muscle protein turnover and signalling, but these findings do not establish long-term tissue preservation during ketogenic weight-loss diets. Supervised very-low-energy ketogenic diets generally produce weight loss composed predominantly of fat mass; however, comparative meta-analyses report small yet larger reductions in FFM or lean body mass than non-ketogenic regimens. Because most studies used hydration-sensitive compartment methods rather than direct anatomical imaging of skeletal muscle, the contributions of glycogen, water, and non-muscle lean tissues remain uncertain. Energy deficit, protein intake, resistance exercise, baseline adiposity, age, metabolic health, and training status may modify the observed response. **Conclusions:** Current evidence is insufficient to determine whether ketosis itself has either a protective or harmful effect on contractile skeletal muscle during weight loss. Ketogenic diets may be effective short-term options for selected adults with obesity, particularly within supervised protocols, but adequate protein, resistance exercise, and monitoring of strength and physical function remain the best-supported muscle-preserving strategies.

## 1. Introduction

Obesity is a chronic, heterogeneous disease. Clinically meaningful weight loss and reduction of excess adipose tissue can improve cardiometabolic and functional complications and quality of life [1], but weight loss is commonly accompanied by some reduction in fat-free mass (FFM), regardless of the strategy used [2,3]. FFM, often used interchangeably with lean body mass (LBM), comprises all non-fat components, including water, glycogen, protein, organs, bone mineral, and the fat-free component of adipose tissue. Therefore, it is not synonymous with contractile skeletal muscle [4,5]. Dual-energy X-ray absorptiometry (DXA) estimates lean soft tissue (LST), whereas anatomical skeletal muscle is more directly quantified with magnetic resonance imaging (MRI) or computed tomography (CT) [5]. Strength and physical function are distinct muscle health indicators that correlate with health outcomes more strongly than muscle mass [6,7], and are particularly important in older adults and people with sarcopenic obesity [6]. Increasing use of potent incretin-based medications has turned the spotlight on body composition during weight reduction [8], but reported lean-compartment changes vary with the magnitude of weight loss, measurement modality, and use of exercise [2,3].

Ketogenic diets are characterised by sufficient carbohydrate restriction to induce nutritional ketosis, shifting fuel use toward fatty acids and ketone bodies [9]. In weight-loss settings, ketosis can modestly attenuate hunger, while ketone bodies act as signalling metabolites with plausible anabolic or anti-catabolic effects in skeletal muscle [10,11,12,13]. Clinical interventions nevertheless differ widely in energy intake, protein content, duration, exercise exposure, achieved ketonaemia, and measurement timing [9,14]. Ketosis alone therefore cannot be assumed to preserve muscle or FFM.

Previous reviews have examined ketogenic diets in relation to obesity treatment, overall body composition, or athletic performance. Bachar and Birk summarised ketogenic obesity interventions monitored by ketone markers and highlighted variability in adherence, macronutrient composition, duration, and lean-body-mass outcomes [15]. Meta-analyses have estimated average changes in FFM or LBM, but these syntheses have generally not integrated acute human tracer and muscle-biopsy evidence with direct anatomical and functional outcomes while explicitly separating ketone exposure from energy deficit, protein intake, exercise, and glycogen–water-related measurement shifts. The present review addresses this interpretive gap rather than re-estimating the overall weight-loss efficacy of ketogenic diets. It integrates mechanistic and clinical evidence to ask whether signalling attributed to ketosis translates into preservation of lean compartments, skeletal muscle, or muscle function during intentional weight loss. It examines ketogenic-diet definitions, protein-turnover mechanisms, clinical body-composition and functional outcomes, phenotype-dependent modifiers, and practical countermeasures.

## 2. Materials and Methods

A structured narrative search of MEDLINE via PubMed was conducted from database inception to 14 August 2026, with reporting guided by SANRA [16]. Two core searches examined: (1) ketogenic dietary interventions in weight-loss or obesity contexts with body-composition, skeletal-muscle, strength, physical-performance, or protein-metabolism outcomes; and (2) ketone bodies or exogenous ketones in relation to skeletal-muscle protein synthesis, breakdown, turnover, leucine kinetics, nitrogen balance, and mTOR/FOXO3a signalling. A targeted supplementary search examined ketone-related AMPK signalling in skeletal muscle. Exposure terms included “ketogenic diet”, “very-low-calorie ketogenic diet”, “very-low-energy ketogenic diet”, “VLCKD”, “VLEKD”, “VLEKT”, “nutritional ketosis”, “ketone bodies”, “beta-hydroxybutyrate”, “beta-hydroxybutyric acid”, “3-hydroxybutyrate”, “3-hydroxybutyric acid”, “acetoacetate”, “acetoacetic acid”, “ketone monoester”, “ketone diester”, “ketone ester”, and “exogenous ketones”. Weight-context terms included obesity, overweight, weight loss, weight reduction, and energy or caloric restriction. Additional searches addressed glycogen- and hydration-related variation in DXA lean estimates and combinations of ketogenic interventions or exogenous ketones with incretin-based therapy. Full search strings and retrieval counts are provided in Supplementary Table S1.

Priority was given to systematic reviews and meta-analyses, randomised controlled trials, prospective intervention studies, and clinical guidelines in adults (Supplementary Table S2). Human tracer and muscle-biopsy studies were included to examine muscle protein turnover and intracellular signalling. Preclinical studies were used to clarify biological mechanisms or phenotype-specific hypotheses when corresponding human evidence was absent or limited. For the core clinical synthesis, ketogenic interventions were required to be characterised by prescribed carbohydrate intake, macronutrient distribution, measured ketonaemia, or use of a recognised very-low-energy ketogenic protocol. Confirmation of achieved nutritional ketosis was recorded when available but was not required, because many clinical studies did not report circulating ketone concentrations.

Non-ketogenic low-carbohydrate studies were not treated as primary evidence of a ketosis-specific effect. However, studies of broader carbohydrate restriction, conventional weight loss, protein intake, resistance exercise, incretin-based therapy, metabolic bariatric surgery, chronic pain, and nutritional safety were included when they provided relevant mechanistic, methodological, or clinical context. Case reports and unsupported opinion pieces were excluded as primary evidence. Outcomes of interest included body composition, anatomical skeletal-muscle measures, strength or physical performance, protein metabolism and signalling, dietary adherence, exercise feasibility, and safety or tolerability. English-language full-text reports were included.

The two core searches returned 325 and 192 records, respectively. The initial long-form AMPK query returned zero records. Because indexed articles were identifiable that contained the constituent ketone-body, skeletal-muscle, and AMPK concepts, the targeted search was repeated using the shorter string reported in Supplementary Table S1; it returned 19 records. Supplementary measurement and incretin searches returned 53 and 12 records, respectively. Reference lists of eligible reviews, meta-analyses, position statements, and key primary studies were searched backward, and forward PubMed citation searches were conducted from six mechanistic anchor studies; 144 unique records were identified through forward citation searching.

MEDLINE via PubMed was selected a priori because the review question centred on biomedical, clinical, and mechanistic evidence and the database indexes the principal human metabolism, obesity, nutrition, and skeletal-muscle literature. Backward and forward citation searching was used to broaden retrieval, but use of a single bibliographic database remains a limitation. The broader phrases “very-low-carbohydrate diet” and “carbohydrate-restricted diet” were examined in supplementary scoping and citation searches but were not used to define the core ketogenic exposure, because carbohydrate restriction alone does not establish nutritional ketosis. When reviews and original studies addressed the same question, recent systematic reviews or meta-analyses were used for overall estimates, while original controlled trials, tracer studies, biopsy studies, and imaging studies were cited for design-specific or mechanistic claims; the two evidence levels were not treated as independent replications of the same data.

A narrative synthesis was selected because the evidence comprises non-equivalent exposures and outcomes: endogenous nutritional ketosis, exogenous ketones, very-low-energy, ad libitum, and eucaloric diets, acute molecular endpoints, longitudinal body composition, strength, and clinical safety. Studies were therefore interpreted within their original clinical context rather than pooled conceptually as a single ketogenic exposure. No new meta-analysis or formal certainty-of-evidence assessment was performed.

For each cited clinical study, the body-composition term used by the investigators and the measurement modality were recorded, because FFM/LBM, DXA-derived LST, and anatomical skeletal muscle are not interchangeable constructs [5]. Original study terminology is retained when results are reported, while interpretation follows the operational distinctions below. DXA-derived LST or study-reported FFM/LBM are not relabelled as skeletal-muscle mass, and early changes during carbohydrate restriction are interpreted in light of concurrent glycogen and fluid shifts [17,18].

Study identification and interpretation were performed as a structured narrative-review process rather than duplicate systematic screening. Exclusion reasons were not prospectively logged at record level, and no independent duplicate risk-of-bias assessment or formal certainty grading was performed. To avoid implying a PRISMA systematic review, retrieval counts, eligibility criteria, evidence groups, and prioritisation rules are reported instead of a reconstructed flow diagram. These features should be considered when interpreting the synthesis. 

**Figure 1:**
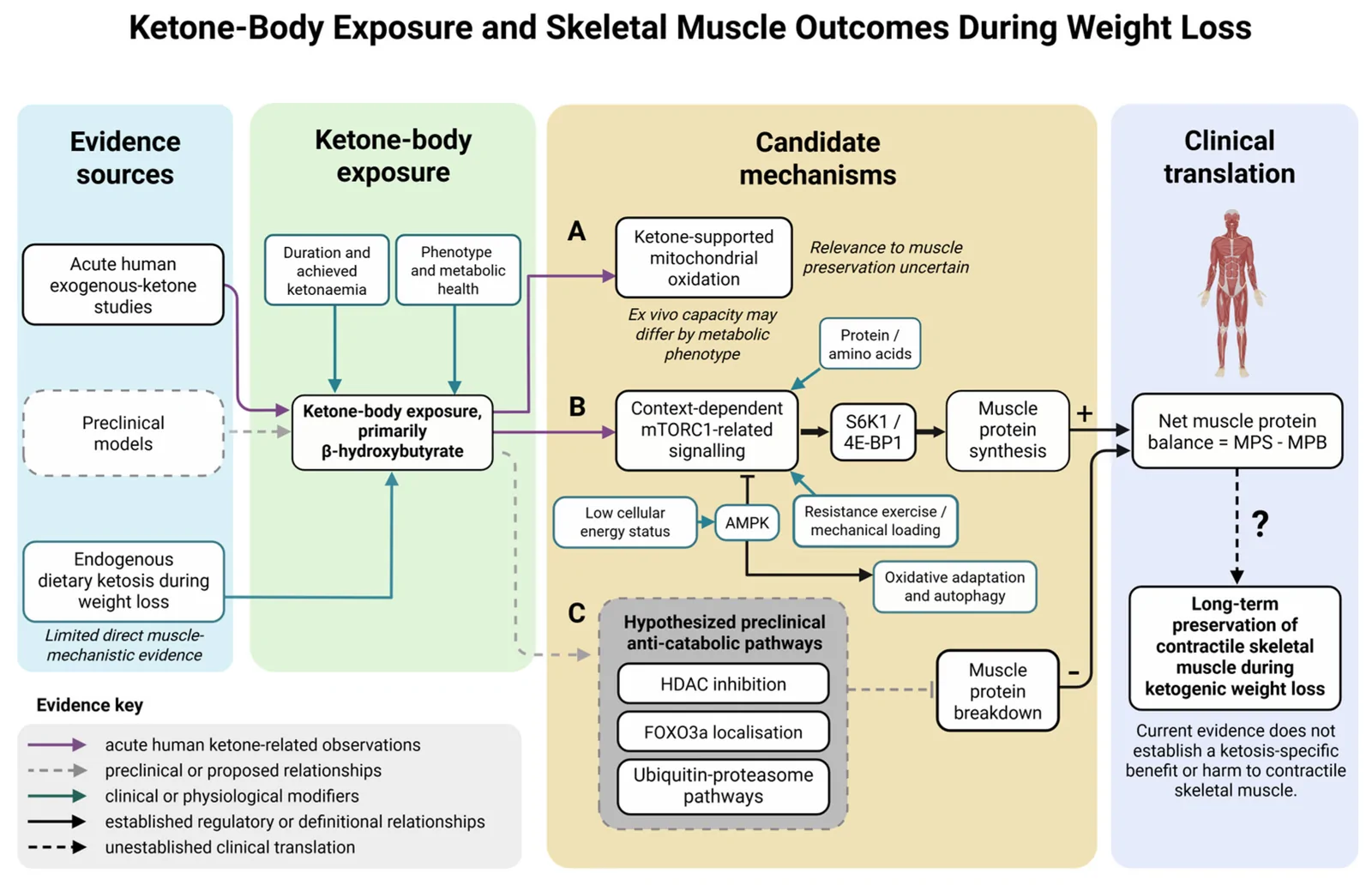
Evidence sources, candidate mechanisms, and clinical translation of ketone-body exposure in skeletal muscle during weight loss. Evidence concerning the effects of ketone bodies on skeletal muscle derives predominantly from acute human studies using exogenous ketones and from preclinical models; direct muscle-mechanistic evidence obtained during endogenous dietary ketosis and weight loss remains limited. The duration of exposure, achieved ketonaemia, phenotype, and metabolic health may influence ketone-body availability, tissue utilization, and downstream responses. (A) Ketone bodies can support mitochondrial oxidation, although ex vivo oxidative capacity may differ according to metabolic phenotype and the relevance of this pathway to muscle preservation remains uncertain. (B) Acute human studies indicate context-dependent relationships between ketone-body exposure and mTORC1-related signalling. mTORC1 regulates MPS through downstream effectors including S6K1 and 4E-BP1. Protein and amino-acid availability and resistance exercise or mechanical loading are shown as established physiological modifiers rather than ketosis-specific effects. Low cellular energy activates AMPK, which inhibits mTORC1-related signalling and contributes to oxidative adaptation and autophagy. (C) HDAC inhibition, altered FOXO3a localization, and modulation of ubiquitin–proteasome pathways have been proposed from preclinical evidence as possible anti-catabolic mechanisms affecting MPB; these relationships have not been established clinically. Net muscle protein balance is represented as MPS minus MPB. The + and − signs indicate the respective positive and negative contributions of MPS and MPB to net balance and do not denote an overall beneficial or harmful clinical effect. Acute changes in signalling or protein turnover cannot be assumed to produce long-term preservation of contractile skeletal muscle. The question mark (?) indicates that current evidence does not establish a ketosis-specific benefit or harm to contractile skeletal muscle during weight loss. Solid purple arrows indicate acute human ketone-related observations; grey dashed arrows and outlines indicate preclinical or proposed relationships; teal arrows and outlines indicate clinical or physiological modifiers; solid black arrows indicate established regulatory or definitional relationships; and the black dashed arrow indicates unestablished clinical translation. T-shaped line endings indicate inhibition. Abbreviations: 4E-BP1, eukaryotic translation initiation factor 4E-binding protein 1; AMPK, AMP-activated protein kinase; FOXO3a, forkhead box O3a; HDAC, histone deacetylase; MPB, muscle protein breakdown; MPS, muscle protein synthesis; mTORC1, mechanistic target of rapamycin complex 1; S6K1, ribosomal protein S6 kinase 1. Created in BioRender. Conte, C. (2026) https://BioRender.com/jv2oipt. Accessed on 4 September 2026.

**Figure 2:**
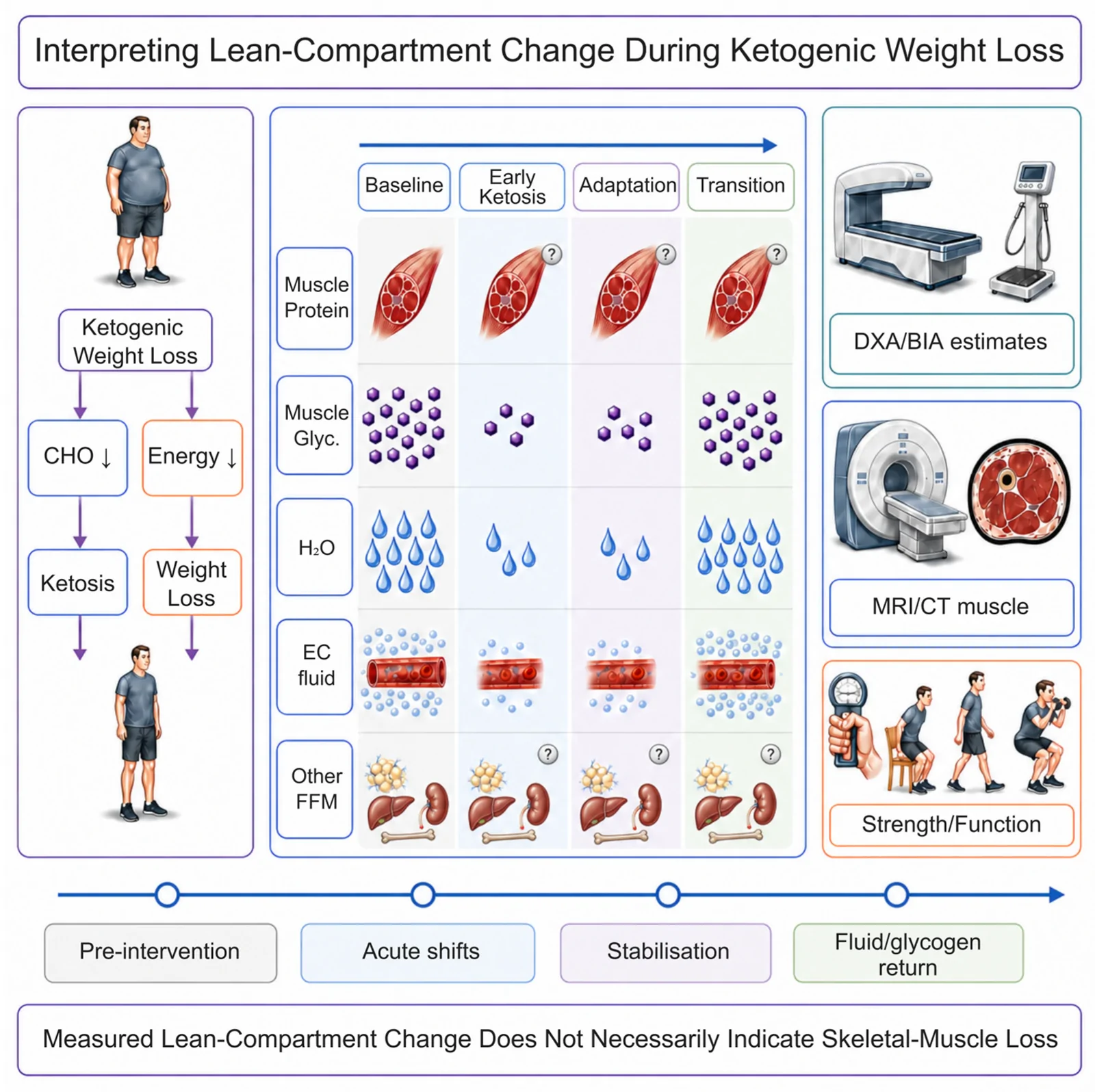
Interpreting lean compartment change during ketogenic weight loss. The left panel separates two related but distinct components of a ketogenic weight-loss intervention: carbohydrate restriction promotes nutritional ketosis, whereas reduced energy intake drives weight loss. The central matrix presents four conceptual stages: baseline, early ketosis, adaptation, and transition. Transition denotes the period following carbohydrate reintroduction. Muscle glycogen, intracellular and glycogen-associated water, and extracellular fluid may decrease during early ketosis, remain lower during adaptation, and recover partly during transition. These changes can alter DXA- or BIA-derived lean-compartment estimates without representing equivalent changes in contractile skeletal-muscle protein. Question marks in the muscle-protein row indicate that the direction and magnitude of muscle-protein change are not established. Other components of FFM include organ tissues, bone mineral, connective tissue, and the fat-free component of adipose tissue. The reduction in the adipose-tissue icon represents the contribution of adipose-tissue loss to measured FFM, whereas question marks indicate that changes in the other depicted components are insufficiently characterised. The number of glycogen symbols, water droplets, and extracellular-fluid symbols indicates the relative direction of the illustrated changes rather than their numerical magnitude. The right panel distinguishes whole-body compartment estimates from anatomical and functional outcomes. DXA estimates LST, whereas BIA estimates FFM; MRI and CT more directly quantify anatomical skeletal muscle. Strength and physical-performance tests assess separate functional domains. The framework concerns interpretation of human clinical body-composition measurements; mechanistic and preclinical evidence is presented separately in Figure 1. Base graphical elements were generated using Illustrae, a generative artificial-intelligence tool, and were subsequently selected, edited, labelled, and scientifically reviewed by the authors. Abbreviations: BIA, bioelectrical impedance analysis; CHO, carbohydrate; CT, computed tomography; DXA, dual-energy X-ray absorptiometry; EC, extracellular; FFM, fat-free mass; glyc., glycogen; H_2_O, water; LST, lean soft tissue; MRI, magnetic resonance imaging.

BioRender AI (BioRender, Toronto, ON, Canada; last accessed on 4 September 2026) was used to generate an initial schematic layout for Figure 1. The scientific content, pathway relationships, labels, and final design were subsequently reviewed and manually revised by the authors. Illustrae (Illustrae Ltd, Eastbourne, UK; last accessed on 25 August 2026) was used to generate base graphical elements for Figure 2 from author-provided prompts. These elements were subsequently selected, edited, annotated, and scientifically reviewed by the authors. The authors take full responsibility for the content of both figures.

## 3. Overview of Ketogenic Diets

A ketogenic diet is a dietary pattern intended to produce nutritional ketosis, commonly defined as a plasma β-hydroxybutyrate (BHB) concentration above 0.5 mmol/L [9]. In adults, this usually requires carbohydrate intake below ~50 g/day or 10% of energy, although the threshold varies with energy expenditure, protein intake, insulin sensitivity, and metabolic adaptation [9]. Nutritional ketosis is a regulated physiological state that differs from ketoacidosis, in which ketone accumulation is accompanied by metabolic acidosis [9,19].

The label “ketogenic diet” covers a wide range of dietary regimens [9,14]. The classical diet used for drug-resistant epilepsy generally provides a 3:1 or 4:1 ratio by weight of fat to combined protein and carbohydrate, whereas medium-chain-triglyceride and modified Atkins approaches allow more protein and carbohydrate. In obesity care, food-based low-carbohydrate ketogenic diets should be distinguished from very-low-energy ketogenic diets (VLEKDs), which typically use meal replacements, provide approximately 500–800 kcal/day and less than 50 g carbohydrate/day, and calculate protein provision from ideal or adjusted body weight during an initial “active” ketogenic phase followed by staged food reintroduction [20,21]. Ad libitum ketogenic diets may also induce a spontaneous energy deficit and reduce body weight.

In adults with obesity, medically supervised VLEKDs can produce substantial short-term reductions in weight and fat mass and improve several glycaemic and cardiometabolic markers [14,21]. Longer-term superiority over sustainable energy-restricted alternatives is not consistently established. In type 2 diabetes (T2D), very-low-carbohydrate ketogenic diets (VLCKDs) can improve glycaemic control and body weight over three to six months, but comparative advantages are less consistent at 12 months, and medication adjustment is essential [22,23]. Evidence does not support a general performance advantage in athletes, and higher-carbohydrate comparators may be superior for some high-intensity outcomes [9,24]. These clinical contexts should not be treated as interchangeable.

Ketogenic therapies used for epilepsy or investigated for other conditions can inform mechanisms or safety but do not directly establish lean-compartment preservation during intentional weight loss. The same limitation applies to evidence from exogenous ketones and preclinical models [9,14].

Interpretation of a ketogenic intervention requires, beyond its indication, information on achieved ketonaemia, prescribed and actual energy and protein intakes, exercise exposure, intervention duration, and measurement timing. Interpretation also depends on whether the body-composition method estimates a molecular compartment, as with densitometry, BIA, or DXA-derived LST, or directly segments anatomical tissue, as with MRI or CT (Table 1).

## 4. Mechanisms Relevant to Lean Mass Preservation

Negative energy balance can suppress muscle protein synthesis and attenuate gains in lean mass from resistance exercise [25,26]. Carbohydrate restriction lowers circulating insulin and raises glucagon, thereby increasing lipolysis, hepatic fatty-acid oxidation, and production of the ketone bodies acetone, acetoacetate, and BHB [9]. During negative energy balance, net muscle protein balance reflects the interaction between protein synthesis and breakdown. Adequate protein intake and resistance exercise support muscle protein synthesis, but severe energy deficit can attenuate these anabolic responses. Any direct effect of ketone bodies must therefore be interpreted within this broader nutritional and mechanical context [25]. Furthermore, acute changes in DXA-derived LST during carbohydrate restriction may partly reflect changes in muscle glycogen and associated water rather than equivalent changes in muscle-protein mass [17,18].

Ketone bodies can interact with anabolic signalling, but the available human evidence derives largely from acute experiments using exogenous ketones [13,27,28]. During recovery from exercise, co-ingestion of a ketone ester with a carbohydrate–protein drink increased phosphorylation of the mTORC1 downstream targets S6K1 and 4E-BP1 relative to an energy-matched control drink containing the same carbohydrate and protein [13]. Parallel experiments in C2C12 myotubes suggested that ketone bodies can potentiate leucine-stimulated protein synthesis. In 36 healthy, recreationally active young men, acute ingestion of a ketone monoester, 10 g whey protein, or both stimulated post-ingestion myofibrillar protein synthesis above the overnight postabsorptive baseline, without significant differences between treatments or in conventional mTORC1 phosphorylation responses [28]. A companion analysis of the same trial demonstrated treatment-related changes in mTOR-associated intracellular trafficking and protein colocalisation, with the most sustained effects observed after ketone monoester and whey-protein co-ingestion [27]. mTORC1 and AMP-activated protein kinase (AMPK) should be interpreted as regulatory nodes rather than direct proxies for tissue accretion. mTORC1 integrates amino-acid, insulin, and mechanical signals to regulate mRNA translation [29], whereas AMPK senses low cellular energy and can restrain mTORC1 while promoting oxidative adaptation and autophagy [30]. During ketogenic weight loss, energy deficit and exercise may influence both pathways independently of circulating ketones [31,32,33]. Direct human evidence that endogenous nutritional ketosis produces a sustained AMPK-mediated muscle-sparing response is lacking. Conversely, transient phosphorylation or altered intracellular trafficking after an exogenous ketone dose does not establish long-term preservation of skeletal muscle during dietary ketosis or ketogenic weight loss [34,35,36].

Other controlled acute human experiments reinforce this uncertainty. In eight young men studied during a model combining endotoxaemia, fasting, and immobilisation, coadministration of BHB with beta-lactoglobulin reduced both muscle protein synthesis and breakdown relative to beta-lactoglobulin alone but did not improve net phenylalanine balance [37]. In ten healthy men, local femoral-artery BHB infusion did not significantly improve net leg phenylalanine balance compared with the contralateral placebo-infused leg, although systemic spillover reduced exposure separation [38]. These studies do not establish benefit or harm during dietary weight loss, but they argue against interpreting acute ketone exposure as uniformly anabolic.

An important limitation of the acute human evidence described above is that it derives predominantly from young, metabolically healthy participants. Metabolic disease may affect both ketone availability and tissue utilisation. During a 24-h fast, hepatic ketogenesis declines as hepatic steatosis and glycaemia increase [39], whereas a short-term hypocaloric ketogenic diet may increase hepatic ketogenesis in adults with overweight or obesity and metabolic dysfunction-associated steatotic liver disease (MASLD) [40]. These hepatic findings demonstrate context-dependent adaptation in ketone production. Using high-resolution respirometry in permeabilised vastus lateralis fibres, Zweck et al. found that ketone-body-supported oxidative phosphorylation capacity was approximately 30% lower in participants with T2D than in glucose-tolerant controls. The contribution of ketone-body-linked respiration relative to maximal coupled NADH- and succinate-supported oxidative phosphorylation was also approximately 50% lower. These ex vivo findings indicate impaired mitochondrial utilisation of ketone bodies in skeletal muscle in T2D, but they do not establish impaired ketone-body signalling, muscle protein synthesis, or proteolysis. They therefore raise, rather than resolve, the possibility that metabolic responses to ketones may differ in insulin-resistant populations [41].

Anti-catabolic effects have also been reported during experimentally induced inflammatory stress. In a randomised crossover study of ten healthy men undergoing lipopolysaccharide-induced endotoxaemia, intravenous 3-hydroxybutyrate reduced basal and insulin-stimulated net forearm phenylalanine release by more than 70% and decreased whole-body phenylalanine-to-tyrosine conversion, an index of protein degradation [42]. Forearm phenylalanine appearance and disappearance were both markedly reduced, indicating suppression of both protein breakdown and synthesis, with the reduction in breakdown predominating. Consistent with reduced synthesis, skeletal-muscle eIF2α phosphorylation increased, whereas the reduction in S6K phosphorylation did not reach statistical significance; measured markers of protein breakdown were unchanged.

In mice, oral administration of a ketone diester attenuated skeletal-muscle wasting in a metastatic cancer-cachexia model and reduced catabolism after lipopolysaccharide administration [11]. The authors proposed that ketone-mediated histone deacetylase inhibition, altered FOXO3a nuclear localisation, and modulation of ubiquitin–proteasome pathways might contribute, but these mechanisms were not established causally. These findings are hypothesis-generating for obesity treatment because acute endotoxaemia, cancer cachexia, and severe nutrient deprivation differ fundamentally from a protein-adequate weight-loss intervention, and both studies examined exogenous ketone administration rather than a ketogenic diet [11,42].

Human evidence for a nitrogen-sparing effect of ketones appears to be context dependent. In nine healthy participants, intravenous BHB reduced whole-body leucine oxidation by a mean of approximately 30% and increased fractional mixed skeletal-muscle protein synthesis by approximately 10%, without altering whole-body leucine flux, during an acute experiment [12]. In contrast, a four-week metabolic-ward study of 16 women with severe obesity found that an isocaloric, isonitrogenous ketogenic diet providing 600 kcal/day produced a more negative cumulative nitrogen balance and greater whole-body leucine oxidation than a non-ketogenic comparator. Proteolysis, assessed by leucine turnover and urinary 3-methylhistidine excretion, did not differ significantly between diets [43]. Acute responses to intravenous ketone administration therefore cannot be assumed to predict net protein retention during prolonged severe energy restriction.

Appetite regulation represents a clinically important indirect pathway through which ketogenic diets may influence body composition. A systematic review and meta-analysis found that participants reported less hunger during VLEKDs and ketogenic low-carbohydrate diets than would ordinarily be expected during weight loss, although the magnitude and mechanisms of this effect remain uncertain [10]. Ketosis may attenuate the weight-loss-associated rise in ghrelin, but the evidence is less extensive than that for subjective appetite. Improved appetite control could facilitate dietary adherence in some individuals or, under ad libitum conditions, contribute to a greater spontaneous reduction in energy intake, although neither consequence should be assumed without direct measurement [10,44]. Accordingly, when energy and protein intake are neither controlled nor measured, differences in fat loss or FFM cannot be attributed confidently to ketosis itself. Interpretation of FFM changes must also account for carbohydrate-related differences in glycogen and body water [5,14].

A further ambiguity concerns circulating branched-chain amino acids (BCAAs). In a six-day crossover study, Qadri et al. found that a hypocaloric ketogenic diet increased plasma leucine, isoleucine, valine, and BCAA-derived acylcarnitines, whereas an energy-matched non-ketogenic diet did not, despite similar reductions in fat mass [45]. Interpretation is limited because the ketogenic diet provided substantially more protein than the comparator diet (29% vs. 19% of energy), and neither skeletal-muscle protein turnover nor muscle amino-acid uptake was measured. The accompanying increases in isobutyrylcarnitine and isovalerylcarnitine were consistent with increased BCAA oxidation, but the source and physiological significance of the higher circulating BCAAs remain uncertain. These metabolomic findings therefore cannot be interpreted as evidence of muscle sparing or, conversely, of increased muscle protein catabolism.

In summary, acute administration of exogenous ketones can influence protein synthesis and breakdown, but these effects are context dependent and cannot be extrapolated directly to prolonged dietary ketosis. Net protein retention during a ketogenic weight-loss diet remains dependent on the severity of energy restriction, protein intake, mechanical loading, and intervention duration [12,28,43]. Molecular and tracer findings therefore provide a rationale for clinical investigation rather than proof that ketogenic diets preserve skeletal muscle or LST. Available clinical evidence remains heterogeneous in its populations, interventions, and body-composition outcomes [36].

## 5. Clinical Evidence on Body Composition Changes Induced by Ketogenic Diets

Clinical evidence derives from randomised trials, comparative studies, and prospective cohorts that vary substantially in population, energy intake, protein content, achieved ketosis, exercise exposure, duration, and body-composition measurement method. Results must therefore be interpreted by context rather than attributed uniformly to ketosis.

Ketogenic weight-loss interventions generally reduce fat mass, although the magnitude and durability of this effect vary according to the population and treatment protocol. In nine elite male gymnasts, a 30-day non-energy-restricted ketogenic diet reduced body weight and skinfold-derived fat mass, while estimated muscle mass and strength performance did not change significantly [46]. This small study, in which participants continued intensive training and consumed a high-protein diet, is not directly comparable with energy-restricted treatment of obesity. In an uncontrolled study of 20 adults with obesity, a structured, multiphase VLEKD programme produced a mean weight loss of 20.2 kg over four months. Of this change, 16.5 kg was classified as fat mass by DXA, 18.2 kg by multifrequency BIA, and 17.7 kg by air-displacement plethysmography [47]. As the intervention included ketogenic and subsequent food-reintroduction phases, the four-month outcome cannot be attributed exclusively to the period of ketosis.

Reductions in total and visceral fat have also been reported in comparative and longitudinal VLEKD studies [48,49]. However, differences in energy prescription and treatment structure complicate interpretation, and longer-term studies commonly include carbohydrate reintroduction followed by a balanced hypocaloric or maintenance diet. For example, the 6- and 12-month outcomes reported by Basolo et al. followed only one month of VLEKD, a four-week carbohydrate-reintroduction phase, and subsequent balanced hypocaloric treatment [50]. Collectively, these studies support the short-term effectiveness of structured ketogenic programmes for reducing fat mass, but they do not establish an advantage of ketosis independent of energy restriction, protein intake, and the accompanying multidisciplinary intervention.

Comparative evidence does not consistently demonstrate superior preservation of FFM or LBM with ketogenic diets. A meta-analysis of 18 randomised controlled trials found greater reductions with ketogenic than with control diets in FFM (weighted mean difference [WMD], −0.81 kg; 95% CI, −1.32 to −0.30), LBM (WMD, −0.63 kg; 95% CI, −1.21 to −0.06) and fat mass (WMD, −1.40 kg; 95% CI, −2.50 to −0.30) [51]. A later meta-analysis similarly found a small reduction in FFM relative to control diets (WMD, −0.48 kg; 95% CI, −0.73 to −0.23; 21 studies and 744 participants). By contrast, the pooled difference in outcomes reported by the primary studies as muscle mass was not significant (WMD, 0.06 kg; 95% CI, −1.97 to 2.09), although this analysis included only four studies and 106 participants [36].

Among participants undertaking resistance training, ketogenic diets were associated with a 1.26-kg less favourable between-group change in FFM than non-ketogenic diets (95% CI, −1.82 to −0.70) [52]. This estimate should not automatically be interpreted as an equivalent loss of contractile muscle because it may reflect both a reduction in FFM and less FFM gain in ketogenic groups relative to controls. More generally, studies often assessed FFM or LBM using DXA, BIA, air-displacement plethysmography, or other compartment models that are sensitive to changes in glycogen and hydration. Energy and protein intakes and premeasurement glycogen status were not consistently standardised. Moreover, the three meta-analyses include overlapping primary studies and therefore do not constitute wholly independent evidence.

Individual obesity studies sometimes report stabilisation or partial recovery of FFM after an initial ketogenic phase, but their designs limit causal interpretation. In a study by Gomez-Arbelaez et al., the largest early reduction in FFM occurred at maximum ketosis. Multifrequency BIA indicated that much of this change coincided with loss of total body water, which partially recovered as ketosis diminished; muscle strength did not decline [47]. These observations suggest that early changes in hydration contributed to the measured FFM reduction, but they do not exclude some loss of lean tissue. In 17 women with obesity, Basolo et al. found that DXA-derived LST decreased by 5.6% during an initial one-month VLEKD and then remained stable during follow-up [50]. The cohort lacked a concurrent control group, and follow-up comprised carbohydrate reintroduction followed by balanced hypocaloric treatment. As such, the subsequent stability of LST cannot be attributed to continued ketosis.

Small comparative studies provide mixed evidence. In a randomised pilot study of 25 participants, an amino-acid- and whey-supplemented VLEKD did not differ significantly from a very-low-energy comparator in total or regional DXA-derived lean outcomes [53]. In 48 participants receiving an intragastric balloon, a low-calorie ketogenic diet produced a smaller reduction in BIA-derived FFM than a standard low-energy diet over four months (3.55% vs. 14.3%) [54]. However, sample size, protein formulation, hydration-sensitive body-composition measurements, and the concurrent balloon intervention limit generalisability. A six-week controlled hypocaloric study provides a particularly informative comparison. Adults with overweight or obesity received a ketogenic diet with or without BHB salts, while a separately assigned group received an isoenergetic, isonitrogenous low-fat diet. Protein was prescribed at 1.5 g/kg ideal body weight/day (approximately 100 g/day), and body composition was assessed by DXA and MRI. Whole-body lean mass and mid-thigh muscle cross-sectional area decreased over time, but changes did not differ significantly among the ketogenic and low-fat groups, and augmenting ketonaemia with BHB salts did not alter body-composition outcomes [55]. The short duration, small groups, and non-random assignment of the low-fat comparator limit inference, but the findings do not support a ketosis-specific advantage under energy- and protein-matched conditions. An eight-week randomised, double-blind, placebo-controlled trial examined exogenous rather than diet-induced ketosis. Fifty-one adults with overweight or obesity received racemic BHB mineral salts or maltodextrin placebo during modest energy restriction. Body weight decreased in both groups. Fat mass, body-fat percentage, and the lean-to-fat-mass ratio improved from baseline only in the BHB group, but the group-by-time interactions for body-composition outcomes were not significant [56]. The trial therefore does not demonstrate a between-group lean-preserving effect of exogenous BHB.

Overall, structured ketogenic interventions can produce weight loss composed predominantly of fat mass, but a ketosis-specific advantage for preserving contractile skeletal muscle remains unproven (Table 2).

## 6. Population- and Phenotype-Dependent Modifiers of Lean Mass Response

Observed changes in lean compartments vary across populations. Baseline adiposity, age, sex, metabolic health, training status, and concomitant therapies may modify body-composition responses alongside energy restriction and dietary composition.

### 6.1. Baseline Adiposity and Metabolic Phenotype

Baseline adiposity is likely an important modifier of body-composition changes during ketogenic weight-loss interventions, as it is during energy restriction more broadly. In the relationship described by Forbes, initial fat mass is inversely and curvilinearly associated with the proportion of weight change attributable to lean tissue: individuals with greater adiposity generally lose a smaller proportion of weight loss as FFM during energy restriction, whereas leaner individuals tend to lose a larger proportion [60,61]. This general partitioning principle is not specific to ketogenic diets. Less favourable hydration-sensitive FFM outcomes have been reported in normal-weight and resistance-trained participants following ketogenic interventions, although athlete-specific findings are inconsistent and these changes cannot be equated directly with loss of contractile muscle [36,52]. Baseline metabolic phenotype may also modify protein and substrate metabolism during energy restriction. Compared with lean participants, people with obesity exhibited lower whole-body amino-acid and urea fluxes and lower forearm protein breakdown after both an overnight fast and 72 h of fasting, suggesting greater protein conservation [62]. These observations do not establish reduced amino-acid use for gluconeogenesis or a ketosis-specific muscle-sparing effect. Differences between VLEKDs and higher-energy ketogenic protocols may reflect baseline phenotype, energy deficit, protein provision, exercise exposure, and measurement conditions, as well as dietary composition.

### 6.2. Age and Sex as Biological Modifiers

Age and sex are plausible modifiers of lean-tissue responses to ketogenic diets, although direct evidence remains limited and heterogeneous.

Older adults are particularly relevant because ageing is associated with anabolic resistance, including a diminished stimulation of muscle protein synthesis by nutrient intake. A systematic review and meta-analysis of 46 studies involving 1280 healthy adults found modestly lower postabsorptive muscle protein synthesis (standardised mean difference [SMD], −0.167) and postprandial muscle protein synthesis (SMD, −0.348) in older than in younger adults, with heterogeneity particularly evident among postprandial studies [63]. Responses to exercise alone were mixed, whereas the muscle protein synthesis response to exercise combined with protein or amino-acid provision was maintained in most studies. These short-term studies did not examine weight loss and therefore cannot establish whether older adults lose more lean tissue during energy restriction. For older adults with obesity, current European Association for the Study of Obesity guidance recommends moderate energy restriction, protein intake of at least 1.0–1.2 g/kg/day, and structured multicomponent exercise, with therapeutic priorities extending beyond weight loss to preservation of muscle function, physical capacity, and quality of life [64]. Intervention studies indicate that combining energy restriction with aerobic and resistance exercise can mitigate losses of muscle and bone while improving physical function [65]. Whether ketogenic protocols provide additional benefits or risks in older adults, including those with sarcopenic obesity, remains uncertain. The ketogenic-diet meta-analyses discussed above did not provide informative age-stratified estimates, and no adequately powered RCT specifically designed to determine the effects of a ketogenic weight-loss diet on skeletal-muscle mass, strength, and physical performance in older adults has been reported. Preclinical studies suggest that ketogenic regimens can modify age-related skeletal-muscle and neuromuscular phenotypes. In aged male mice, long-term ketogenic feeding was associated with greater gastrocnemius mass and changes in muscle fibre type, mitochondrial characteristics, oxidative-damage markers, and physical performance [66]. In another aged-mouse model, ketogenic feeding improved selected motor outcomes and measures of motor-unit connectivity [67].

According to the International Society of Sports Nutrition, there is “insufficient evidence to determine if a ketogenic diet affects males and females differently,” while recognising “a strong mechanistic basis for sex differences to exist” [9]. This position statement primarily concerns healthy exercising adults and does not establish sex-specific effects in people undergoing obesity treatment. A meta-regression of studies in trained adults identified study-level sex composition as a possible moderator of total body-mass change, whereas the estimate for FFM remained uncertain [24]. Such analyses are exploratory and cannot substitute for trials reporting prospectively specified, sex-stratified outcomes. In mice, ketogenic and moderately low-carbohydrate diets produced sexually dimorphic changes in body weight, adipose tissue, insulin, ghrelin, and fibroblast growth factor 21; for example, the ketogenic diet reduced body weight and subcutaneous fat in males but not females [68]. These findings demonstrate biological sex differences in the response to carbohydrate restriction but do not establish differential effects on muscle preservation in humans.

Whether sex, menopausal status, or sex-hormone exposure interacts with endogenous ketosis to modify skeletal-muscle protein turnover during weight loss remains unknown. Trials should report prespecified sex-stratified body-composition, strength, and functional outcomes.

### 6.3. Type 2 Diabetes and Incretin-Based Therapies

Underlying metabolic disease and concomitant pharmacological therapies may modify responses to ketogenic interventions and the interpretation of body-composition outcomes. People with T2D often lose less weight with obesity pharmacotherapy than those without diabetes, for reasons that may include metabolic flexibility, concomitant medications, and social determinants of health [69]. Whether the same factors alter weight-loss partitioning during ketogenic interventions is unknown. Diabetes-associated abnormalities in muscle mass, quality, and function make anatomical and functional outcomes particularly important [70]. As discussed in Section 4, ex vivo skeletal muscle from people with T2D showed impaired ketone-body-supported mitochondrial oxidative capacity, but this does not establish impaired ketone signalling or muscle preservation during nutritional ketosis [41].

Ketogenic diets can improve glycaemic and other metabolic outcomes in people with T2D, but evidence concerning skeletal muscle remains insufficient. Exploratory post hoc analyses reported in a perspective article suggested that, within a non-randomised continuous-care intervention incorporating carbohydrate restriction and nutritional ketosis, greater ketosis or dietary adherence was associated with a slightly smaller reduction in lower-extremity lean mass relative to total weight loss; total LBM did not differ significantly across adherence categories [71]. These findings are hypothesis-generating because the study was not an RCT, the intervention included multiple components, and DXA-derived lower-extremity lean mass is not equivalent to contractile skeletal-muscle mass. In a systematic review and meta-analysis of RCTs lasting at least six months in adults with prediabetes or T2D, only one trial reported FFM, with no significant difference between the VLCKD and comparator diets [22]. No adequately powered RCT has evaluated anatomical skeletal-muscle mass, strength, and physical performance as primary outcomes of a ketogenic weight-loss intervention in people with T2D.

Evidence concerning the combination of ketogenic dietary interventions with incretin-based pharmacotherapy is also preliminary. In a short-term, non-randomised real-world study of people with obesity and no diabetes, participants receiving a meal-replacement VLEKD plus liraglutide lost more weight than those receiving VLEKD alone, but body composition, strength, and physical performance were not assessed [72]. The non-randomised design also precludes confident attribution of the difference to a synergistic interaction between liraglutide and ketosis. A retrospective case series described seven adults with overweight or obesity who completed a six-month clinician-supervised programme combining an individualised whole-food ketogenic diet with semaglutide at doses up to 1.0 mg/week. Mean BIA-estimated skeletal-muscle mass decreased by 1.2 kg during substantial weight loss, and one participant gained lean tissue [73]. The small uncontrolled sample, individualised diet and drug exposure, and hydration-sensitive measurement method prevent attribution of the body-composition pattern to ketosis, semaglutide dose, or their combination. No controlled clinical trial has established that a ketogenic diet or exogenous ketones preserve anatomical muscle or function during incretin-based therapy.

In male mice with diet-induced obesity and glucose intolerance, three weeks of oral ketone-ester supplementation attenuated semaglutide-associated losses of whole-body lean mass, individual skeletal-muscle mass, and myofibre size, while improving selected measures of strength and exercise performance [74]. Ketone supplementation also contributed energy and attenuated total weight loss, although fat-mass loss was reportedly similar between the semaglutide groups. These findings warrant clinical testing, but neither ketogenic dietary interventions nor exogenous ketones can currently be considered established muscle-preserving adjuncts to incretin-based therapy.

Collectively, changes in lean compartments during ketogenic interventions must be interpreted in relation to the clinical and metabolic characteristics of the population studied (Table 3). These modifiers, together with energy deficit, protein provision, exercise, intervention duration, and body-composition methodology, help explain the seemingly discordant mechanistic and clinical evidence discussed next.

## 7. Reconciling Mechanism and Clinical Evidence

Acute ketone experiments and preclinical models provide a plausible anabolic or anti-catabolic rationale, whereas comparative clinical studies do not demonstrate consistent preservation of FFM and rarely measure anatomical skeletal muscle directly. This is not necessarily contradictory, because the studies evaluate non-equivalent exposures and outcomes. Acute exogenous ketone administration cannot be equated with prolonged endogenous ketosis during energy restriction, and molecular signalling or short-term protein turnover cannot predict skeletal-muscle mass or function. Differences in energy deficit, protein intake, mechanical loading, phenotype, duration, and hydration-sensitive measurements further prevent attribution to ketosis. Current evidence therefore establishes neither a ketosis-specific benefit nor direct harm to contractile skeletal muscle. The relationship between ketone-body mechanisms, clinical modifiers, measurement methods, and functionally relevant outcomes is summarised in Figure 1.

Dietary protein is a particularly important source of confounding. Protein intake is not consistently matched or adequately reported in comparisons of ketogenic and non-ketogenic diets, and studies included in clinical reviews vary in both protein and energy prescription [44,52]. During energy restriction, higher protein intake can attenuate loss of FFM, although the magnitude of benefit depends on the energy deficit, baseline phenotype, physical activity, and intervention duration [25,75]. In a meta-regression, protein intake above 1.05 g/kg/day was associated with 0.60 kg greater FFM retention, increasing to 1.21 kg in interventions lasting more than 12 weeks [75]. More directly, during four weeks of 30% energy restriction in recreationally active women, approximately 2.0–2.2 g protein/kg/day preserved DXA-derived lean tissue, whereas lower protein intake with either placebo or exogenous ketones did not [34]. Thus, when protein intake differs between dietary groups, any apparent preservation of FFM cannot be attributed specifically to ketosis.

Interpretation is further complicated by inconsistent intervention classification. Diets labelled “ketogenic” vary substantially in prescribed carbohydrate intake, energy and protein content, and adherence, while circulating ketones are not always measured [44]. Consequently, some trials compare verified nutritional ketosis with a non-ketogenic diet, whereas others compare dietary prescriptions whose achieved metabolic states remain uncertain. This exposure heterogeneity limits both between-study comparisons and pooled estimates of a ketosis-specific effect.

Glycogen and associated water are important sources of variability in body-composition measurements. An older metabolic balance study provides direct evidence that early scale-weight differences need not represent proportional tissue loss. Six adults with obesity completed 10-day periods of starvation, an 800-kcal mixed diet, and an isocaloric 800-kcal ketogenic diet. Weight loss was greater during the ketogenic than the mixed diet, but the additional loss was predominantly water; protein represented 3.8% and 3.4% of weight lost, respectively [76]. The study was small, brief, and used historical balance methods, but it supports separating early water loss from protein loss rather than inferring muscle preservation or wasting from body weight alone. Experimental manipulation studies provide a plausible scale for non-protein changes in DXA-derived lean estimates over periods too short for substantial changes in muscle protein. In trained cyclists, glycogen loading increased whole-body DXA lean mass by approximately 2–3% and leg lean mass by 2.6–3.1%, while glycogen depletion reduced leg lean mass by 1.4% [17]. In 12 active men, exercise and thermal dehydration reduced body mass by 1.93 kg and DXA-derived lean tissue by 1.69 kg; subsequent rehydration and glycogen supercompensation increased body mass by 2.53 kg and lean tissue by 2.36 kg within 48 h [18]. These experimental conditions are not equivalent to ketogenic weight loss and cannot be used to correct individual trials. Nevertheless, these experimentally induced changes in DXA-derived lean estimates were similar to or greater than the average between-diet FFM differences reported in meta-analyses (approximately 0.48–0.81 kg). They demonstrate that glycogen and hydration shifts can overlap in magnitude with small pooled differences, but they do not quantify the contribution of these components in ketogenic trials or exclude concomitant tissue loss.

A similar pattern was observed in resistance-trained men after a ketogenic intervention. Wilson et al. reported a 4.8% increase in DXA-derived LBM between weeks 10 and 11 after rapid carbohydrate reintroduction in the ketogenic group [77]. An increase of this magnitude within one week is unlikely to represent new contractile tissue and is most plausibly explained, at least in part, by restoration of glycogen and associated water. Collectively, these observations indicate that early changes in FFM or LBM during carbohydrate restriction may partly reflect non-protein components.

Measurement timing and standardisation are therefore critical. Short interventions may disproportionately capture the acute effects of glycogen depletion and fluid redistribution. Longer follow-up can reduce the influence of the initial transition, but comparisons remain vulnerable when carbohydrate intake (and consequently glycogen status) differs systematically between groups. Serial measurements obtained under standardised dietary, hydration, activity, and glycogen-related conditions are preferable. A planned carbohydrate-reintroduction assessment may help estimate the reversible component of an apparent lean-tissue change, but it should be interpreted separately from body composition during sustained ketosis. Even long-duration studies remain difficult to interpret when the dietary protocol changes over time or when a concurrent energy- and protein-matched control is absent. These time-dependent compartment changes and the corresponding approach to body-composition and functional assessment are summarised schematically in Figure 2.

Ad libitum ketogenic diets can produce spontaneous energy restriction. In a systematic review of physically active adults, energy balance estimated from changes in body composition averaged approximately −339 kcal/day in ketogenic groups compared with +5 kcal/day in control groups [59]. Appetite responses, dietary restrictiveness, food choice, and accompanying differences in protein intake may all contribute. Ad libitum comparisons therefore evaluate the ketogenic dietary strategy as a whole but cannot isolate effects of carbohydrate restriction or ketosis from those of energy and protein intake.

As detailed in Table 1 and Figure 2, FFM/LBM, DXA-derived LST, and anatomical skeletal muscle are not interchangeable, and strength and physical performance represent distinct outcomes [5]. The clinical meaning of a small between-diet difference in FFM cannot be inferred from mass alone. In the meta-analysis by Wang et al., ketogenic diets were associated with a small additional reduction in FFM relative to control diets, whereas outcomes reported as muscle mass and measures of squat and bench-press strength did not differ significantly [36]. However, the muscle-mass estimate was based on only four studies and 106 participants. The functional significance of this small mean difference is unknown. No trial has established phenotype-specific thresholds linking FFM change to disability, falls, or loss of independence during a ketogenic diet.

Muscle strength and physical performance are more closely associated with clinical outcomes than muscle mass alone [6,7]. This distinction is reflected in the European Working Group on Sarcopenia in Older People 2 (EWGSOP2) consensus, which uses low muscle strength to identify probable sarcopenia, low muscle quantity or quality to confirm the diagnosis, and poor physical performance to indicate severity [6]. Consistent with this distinction, evidence from non-ketogenic weight-loss interventions indicates that absolute strength can decline while relative strength and physical performance improve [78], and that upper- and lower-limb strength may not change in parallel [79]. The clinical meaning of a given FFM change depends on baseline phenotype and function. In a younger adult with severe obesity and preserved strength and performance, a small FFM reduction during substantial weight loss cannot by itself establish clinically meaningful skeletal-muscle loss [5,60,61]. In an older adult with low strength or sarcopenia risk, the same absolute change may warrant closer evaluation, because functional reserve may already be limited [64,65]. During incretin-based therapy, available meta-analyses quantify changes in lean compartments but do not establish corresponding impairment in strength or physical performance [2,3]; nor has a ketogenic diet or exogenous ketones been shown clinically to prevent such impairment [72,73,74]. These distinctions support interpreting FFM changes alongside longitudinal measures of strength and physical performance rather than applying an absolute or proportional FFM threshold alone.

Reductions in lean compartments should also be interpreted within the normal physiology of weight loss. Dietary interventions, incretin-based pharmacotherapy, and bariatric surgery all produce some reduction in FFM, although the magnitude and composition vary [80]. Cross-treatment meta-analyses yield divergent proportional FFM- or lean-mass-loss estimates because eligibility criteria, achieved weight loss, intervention composition, exercise exposure, duration, and measurement methods differ [2,3]. Moreover, adipose tissue is not composed exclusively of triglyceride: it contains water, protein, mineral, structural lipids, glycogen, and other fat-free components. Loss of adipose tissue therefore necessarily contributes some mass to the measured reduction in FFM [5].

Overall, current evidence cannot determine whether the molecular actions of ketone bodies translate into clinically meaningful, ketosis-specific preservation of skeletal muscle during weight loss. We identified no long-term trial that compared ketogenic and non-ketogenic weight-loss diets while matching energy and protein intake, documenting achieved ketonaemia, and prespecifying MRI- or CT-derived skeletal muscle together with strength and physical performance. The observed clinical signal reflects the combined effects of energy deficit, protein intake, exercise, population phenotype, dietary protocol, intervention duration, and measurement conditions. Molecular findings therefore provide a rationale for better-controlled clinical trials rather than proof of muscle-preserving benefit.

## 8. Protein Intake, Exercise, and Practical Strategies for Preserving Lean Mass

Across weight-loss interventions, adequate individualised protein intake and resistance exercise are the best-supported available strategies for limiting loss of lean tissue, with resistance exercise also supporting strength, irrespective of the dietary model used [25,81,82]. Across the broader weight-loss literature, protein intakes of approximately 1.2–1.6 g/kg/day, preferably distributed across meals, are associated with greater satiety and better lean-mass retention than lower intakes, but the optimal target and body-weight denominator remain uncertain in obesity [81,83]. Targets should be individualised according to age, adiposity, severity of energy restriction, renal function, comorbidities, and training load. Protein cannot be assumed to fully offset the effects of a severe energy deficit [25,26].

Resistance exercise provides a mechanical stimulus for preserving contractile tissue and strength that protein intake alone cannot reproduce. Across weight-loss trials in people with overweight or obesity, resistance exercise attenuates FFM loss and improves strength, although effects vary with intervention duration and energy deficit [82,84]. It is not a complete safeguard during ketogenic dieting: a meta-analysis of 13 resistance-training trials found a 1.26-kg less favourable between-group change in FFM with ketogenic than with non-ketogenic diets, alongside greater fat loss [52]. Again, differences in hydration, glycogen, energy intake, and protein intake may contribute to this estimate.

Chronic musculoskeletal pain is common in people with obesity and may limit participation in resistance exercise, increasing vulnerability to functional decline [85,86]. Weight management can improve pain in some musculoskeletal conditions, although exercise progression may require individualised, multimodal pain management [87,88,89]. Preliminary dietary evidence does not establish a ketosis-specific analgesic effect. In a small pilot trial, pain improved with both a carbohydrate-restricted whole-food diet and a whole-food comparator. Ketonaemia was modest, pain change was not associated with ketone concentrations, and greater energy restriction and weight loss in the carbohydrate-restricted group prevented attribution to ketosis [90].

Implementation should begin with assessment of diet, weight trajectory, medication use, comorbidities, functional status, and sarcopenia risk [6,91]. When clinically indicated, DXA or existing CT or MRI can complement anthropometry; handgrip strength, sit-to-stand performance, gait speed, and training records help determine whether a compositional change is functionally meaningful. Repeat body-composition measurements should use the same modality under comparable preassessment conditions for hydration, feeding, exercise, and carbohydrate or glycogen status [17,18]. An isolated early decline in DXA-derived LST or BIA-estimated FFM should not be labelled muscle wasting without corroborating longitudinal, anatomical, dietary, or functional evidence.

Diet quality should be preserved within the carbohydrate limit. Carbohydrate-restricted diets may reduce micronutrient intake when food selection or supplementation is inadequate [92]. Emphasising non-starchy vegetables, nuts and seeds, unsaturated fats such as olive oil, fish, and minimally processed protein sources can improve nutrient density within a ketogenic pattern [20,44,91]. Hydration, electrolytes, bowel function, symptoms, and micronutrient adequacy require particular attention during initiation and in very-low-energy protocols [91]. Similar attention to protein intake, nutrient density, resistance exercise, and muscle-function monitoring is recommended for patients receiving incretin-based therapy [93,94].

A medically supervised VLEKD may be reasonable for selected adults with obesity who have a clinically assessed need for substantial short-term weight reduction [20,21]. Additional caution and individualised assessment are warranted in older adults, people with sarcopenic obesity, and those with eating-disorder risk, clinically significant renal or hepatic impairment, or medication-related risks, because direct evidence is limited and vulnerability to adverse compositional or functional outcomes may be greater. In these groups, nutritional adequacy, muscle strength, physical performance, and multidisciplinary clinical management should take precedence over scale weight alone.

## 9. Safety, Tolerability, and Clinical Boundaries

Adverse effects during ketogenic-diet initiation are commonly gastrointestinal or constitutional, although safety and tolerability evidence is protocol- and indication-specific. Findings from classical ketogenic therapy for epilepsy, higher-energy ketogenic diets, and obesity-focused VLEKDs should not be pooled conceptually or extrapolated without regard to age, disease, energy intake, duration, medication use, and clinical supervision. A 2026 systematic analysis of heterogeneous therapeutic ketogenic interventions—not specifically obesity-focused VLEKDs—reported at least one adverse event in 43% of participants. Among the 743 adverse events recorded in interventions with complete counts, 40% were gastrointestinal, and constipation accounted for 23.9% of all events [95]. These estimates should not be generalised directly to a specific obesity protocol. Constipation, nausea, abdominal pain, headache, dizziness, fatigue, and muscle cramps warrant systematic monitoring.

Nutritional adequacy depends on food selection and supplementation rather than carbohydrate quantity alone. Studies of carbohydrate-restricted diets report reductions in intakes of thiamine, folate, magnesium, calcium, iron, and iodine, although the magnitude varied across nutrients and studies and the included evidence did not establish clinical deficiency [92]. These risks reinforce the practical measures described above. In adults with overweight or obesity, available studies have not identified consistent adverse changes in BMD or bone turnover attributable specifically to ketogenic diets. However, small samples, heterogeneous protocols, limited duration, concurrent weight loss, and reliance on surrogate skeletal endpoints preclude firm conclusions, particularly regarding long-term fracture risk [96,97].

Exercise performance is a potential limitation in trained populations. Fat oxidation increases substantially after adaptation, but athletic performance is generally neutral or impaired relative to higher-carbohydrate diets, particularly in high-intensity activities and settings in which exercise economy is important; maximal strength and strength gains are generally similar [9,24]. Any reduction in training volume or quality could indirectly weaken an important muscle-preserving stimulus during weight loss. Athletes should therefore be monitored for performance and training quality rather than assessed by body weight or FFM alone.

Medication and disease-related risks require screening before major carbohydrate or energy restriction [21,91]. Insulin and sulfonylureas may require dose reduction, and antihypertensive agents or diuretics may require review as plasma glucose, blood pressure, and fluid balance change. SGLT2 inhibitors may increase the risk of diabetic ketoacidosis and are considered contraindicated during VLEKD because of the potential for euglycaemic presentations [19,21]. VLEKD is also generally contraindicated during pregnancy or lactation and in people with eating disorders, type 1 diabetes, kidney failure or severe chronic kidney disease, liver failure, unstable cardiovascular disease or a recent major cardiovascular event, and disorders of fatty-acid oxidation. Other medically complex states require individualised specialist assessment rather than extrapolation from uncomplicated obesity cohorts.

Evidence on long-term adherence, safety, and muscle function remains limited, particularly outside structured obesity programmes. In the cited studies, the initial ketogenic phase was followed by carbohydrate reintroduction and subsequent balanced energy restriction. Longer-term outcomes therefore reflect the complete treatment sequence rather than sustained ketosis alone [47,50].

## 10. Limitations of the Evidence Base and Future Research Directions

The evidence base has important design and reporting limitations. Many interventions were small or short, and lean-compartment outcomes were commonly secondary endpoints for which trials were not specifically powered. Ketogenic protocols varied in carbohydrate and energy restriction, protein provision, duration, food quality, and exercise exposure; achieved ketonaemia and actual energy and protein intakes were not consistently documented. Populations ranged from sedentary adults with obesity to athletes and patients treated for non-weight-loss indications. Adherence, attrition, adverse events, and missing body-composition data were variably reported. These differences limit quantitative comparisons and causal attribution to ketosis.

Outcome definition and measurement were also inconsistent: nonspecific “lean mass”, DXA-derived LST, and anatomical skeletal-muscle mass were sometimes conflated, while MRI- or CT-derived measures and functional outcomes were uncommon [2,5]. Cross-modality comparisons involving dietary interventions, incretin-based therapy, and bariatric surgery should also be interpreted cautiously because estimates depend on eligibility criteria, treatment intensity, measurement method, heterogeneity, and analytic assumptions [2,3].

Finally, this review used a structured narrative rather than a systematic-review process. Although search strings, retrieval counts, eligibility criteria, and citation-search routes were documented, study selection and quality assessment were not performed independently in duplicate, record-level exclusion reasons were not prospectively logged, and formal risk-of-bias or certainty grading was not undertaken. The synthesis should therefore be interpreted as an evidence-informed critical appraisal rather than an exhaustive quantitative estimate.

Future randomised controlled trials should be designed specifically to determine whether ketosis modifies skeletal-muscle outcomes during weight loss. They should compare ketogenic and non-ketogenic diets matched for energy and protein intake, document adherence, actual dietary intake, and achieved ketonaemia, and prespecify direct anatomical measures of skeletal muscle, using MRI or CT where feasible, together with standardised measures of strength and physical performance, rather than relying on DXA- or BIA-derived lean compartments alone. Assessments before, during, and after carbohydrate reintroduction would help distinguish acute compartment shifts from tissue change. Trials should be adequately powered and sufficiently long and should report sex-disaggregated results and phenotype-specific outcomes in older adults, people with sarcopenic obesity or T2D, and patients receiving incretin-based therapy.

## 11. Conclusions

Ketogenic diets can produce clinically meaningful short-term weight loss, particularly when delivered as medically supervised very-low-energy protocols for selected adults with obesity. Current evidence is insufficient to determine whether ketosis itself has either a protective or harmful effect on contractile skeletal muscle during weight loss. Some acute human and preclinical studies report context-dependent effects of ketone bodies on protein synthesis and breakdown, but these mechanistic observations have not been shown to translate into superior muscle preservation during ketogenic dietary weight loss. Small additional reductions in DXA-derived lean estimates or BIA-estimated FFM relative to non-ketogenic comparators may be materially influenced by glycogen and water, although the contribution of each component is unknown and direct anatomical muscle data remain sparse. Adequate individualised protein intake, resistance exercise, and monitoring of strength and physical performance remain better-supported strategies for protecting muscle than ketosis itself.

Future trials should match energy and protein intake, document adherence and achieved ketonaemia, standardise conditions affecting hydration and glycogen, and combine whole-body compartment estimates with anatomical and functional outcomes, particularly in older adults, sarcopenic obesity, T2D, and incretin-treated populations. Until such evidence is available, ketogenic diets should be selected according to their overall clinical suitability, safety, and feasibility within a structured treatment pathway rather than an assumed intrinsic ability to spare muscle.

## Figures and Tables

**Table 1:** Operational terminology used in this review.

Term	Level/Measurement	Interpretation in This Review
Fat-free mass (FFM)/lean body mass (LBM)	Molecular compartment Multicomponent models, densitometry, dilution methods, or BIA; sometimes reported from DXA	All non-fat constituents, including water, glycogen, protein, organs, bone mineral, and fat-free adipose tissue. FFM and LBM are generally used interchangeably and are not synonymous with skeletal muscle [5].
Lean mass	Nonspecific term Definition and method vary by study	Retain the source term; do not infer a specific compartment or skeletal muscle without a clear operational definition [5].
Lean soft tissue (LST)/appendicular LST (ALST)	Molecular compartment DXA; ALST is the sum of arm and leg LST	Non-fat, non-bone-mineral soft tissue. ALST is a proxy for appendicular muscle under standardised conditions but includes non-muscle tissues and hydration-sensitive components [5,6].
Skeletal muscle mass	Anatomical tissue MRI or CT; regional ultrasound measures muscle dimensions	Use skeletal-muscle terminology only when muscle is directly segmented or a validated muscle-specific method is reported [5].
Muscle function	Functional/Strength or performance tests	Report strength and physical performance separately from mass because these are complementary clinical domains [6].

Original study terms are retained, but interpretation follows the measurement level and modality.

**Table 2:** Main characteristics of ketogenic dietary approaches and reported body-composition findings.

Ketogenic Approach	Typical Energy Intake	Common Assessment Methods	Reported Fat-Mass Findings	Reported Lean-Compartment Findings	Clinical Interpretation	Evidence Base & Main Limitations	Ref.
VLEKD	Approximately 500–800 kcal/day during the active phase	DXA, MF-BIA, ADP, indirect calorimetry	Marked short-term reductions in total and visceral fat; longer-term outcomes commonly include carbohydrate reintroduction and subsequent non-ketogenic treatment	Early FFM or LST reductions may partly reflect glycogen and body-water depletion; stabilisation or recovery after carbohydrate reintroduction does not establish preservation during ketosis, and anatomical skeletal muscle is rarely measured	Effective short-term obesity treatment; does not establish a ketosis-specific muscle-sparing effect	Prospective cohorts and small comparative/pilot trials; lean outcomes usually secondary; protocol transitions and limited direct muscle imaging.	[47,48,49,50,53,57]
LEKD	Approximately 800–1200 kcal/day	BIA	Trend toward greater fat mass reduction than conventional low-energy diet	Some small trials report less BIA-derived FFM loss than conventional low-energy comparators	Protocol-specific findings require confirmation in adequately powered, energy- and protein-matched trials	Small randomised trial(s), frequently BIA-based; protocol-specific protein provision and treatment context limit generalisability.	[54]
Eucaloric or minimally energy-restricted KD	Maintenance or near-maintenance energy intake	DXA, skinfolds, BIA	Increased lipid oxidation with modest changes in body composition	Hydration-sensitive lean-compartment estimates and strength may remain stable, but outcomes vary by population and method	Useful for separating energy restriction from diet composition only when energy and protein are controlled	Small controlled or athletic studies; short duration; energy control and population vary; hydration-sensitive outcomes common.	[46,58]
Ad libitum ketogenic diet	Variable; often a spontaneous energy deficit	Variable	Fat mass reduction generally associated with spontaneous reduction in energy intake secondary to increased satiety	Meta-analyses report small average FFM reductions; the contributions of spontaneous energy deficit, glycogen, water, and tissue loss are uncertain	Tests the dietary strategy as a whole; cannot isolate a ketosis-specific effect	Heterogeneous trials and systematic reviews; energy and protein often unmatched.	[59]

Abbreviations: ADP, air displacement plethysmography; BIA, bioelectrical impedance analysis; DXA, dual-energy X-ray absorptiometry; FFM, fat-free mass; KD, ketogenic diet; LBM, lean body mass; LEKD, low-energy ketogenic diet; LST, lean soft tissue; MF-BIA, multifrequency bioelectrical impedance analysis; VLEKD, very-low-energy ketogenic diet.

**Table 3:** Population- and phenotype-specific modifiers of lean-compartment responses to ketogenic diets.

Population/Phenotype	Observed Lean-Compartment Response	Relevant Evidence and Interpretive Considerations	Clinical Implication	Quality of Evidence	Ref.
Adults with overweight/obesity	Heterogeneous; predominantly fat-mass loss is common, but comparative FFM preservation is not established	Greater baseline fat mass predicts a lower proportional FFM contribution to weight loss	Do not infer a ketosis-specific advantage; interpret energy deficit, protein, exercise, and measurement timing	Meta-analyses plus heterogeneous interventions; no consistent direct-muscle evidence.	[14,52,60,61]
Lean/trained individuals	Heterogeneous changes in hydration-sensitive FFM; direct anatomical muscle measurements and functional outcomes remain limited	General weight-loss partitioning is influenced by baseline adiposity, while glycogen and hydration can alter compartment-derived FFM estimates. Neither observation establishes a ketosis-specific effect in trained participants	Findings should not be extrapolated to sedentary obesity treatment; monitor training quality and glycogen-sensitive measures	Mostly short trials and systematic reviews; hydration-sensitive FFM; limited MRI/CT and function.	[17,18,24,52,59,60,61]
Older adults	Human evidence insufficient	Anabolic resistance; sarcopenia risk	Prioritize protein adequacy, resistance exercise, strength, and performance monitoring	Indirect human and preclinical evidence; no dedicated muscle-preservation RCT.	[63,64,65]
Men versus women	No established sex-specific FFM or skeletal-muscle response	Sex-dependent hormonal and metabolic responses are plausible but not clinically established	No sex-specific clinical recommendations can currently be made	Position statement, meta-regression, and preclinical evidence; no adequately powered sex-stratified clinical muscle outcomes.	[9,24,68]
T2D/metabolic dysfunction	Insufficient direct evidence for anatomical skeletal muscle or function	Diabetes-associated muscle abnormalities and impaired mitochondrial ketone oxidation may modify responses	Dedicated RCTs with anatomical and functional outcomes are needed	Post hoc observational evidence and very limited RCT body-composition reporting; no powered anatomical/functional endpoints.	[22,41,71]
Patients receiving incretin-based therapies	No clinical evidence of a muscle-sparing combination	Preclinical ketone-ester findings have not been confirmed in controlled human studies	Emerging research question; no clinical recommendation can currently be made; controlled clinical trials are required	One non-randomised clinical study without body composition, one uncontrolled case series using BIA, and one exogenous-ketone mouse experiment; no controlled clinical muscle-preservation trial.	[72,73,74]

Abbreviations: FFM, fat-free mass; LST, lean soft tissue; T2D, type 2 diabetes; VLEKD, very-low-energy ketogenic diet.

## Data Availability

No new data were created or analysed in this study. Data sharing is not applicable to this article.

## Supplementary Materials

The following supporting information can be downloaded at: https://www.mdpi.com/article/10.3390/nu18172933/s1, Table S1. Search documentation. Table S2. Study eligibility and prioritization.

## Abbreviations

The following abbreviations are used in this manuscript:

**Abbreviation**	**Definition**
4E-BP1	Eukaryotic translation initiation factor 4E-binding protein 1
ADP	Air displacement plethysmography
ALST	Appendicular lean soft tissue
AMPK	AMP-activated protein kinase
BCAA	Branched-chain amino acid
BHB	β-hydroxybutyrate
BIA	Bioelectrical impedance analysis
BMD	Bone mineral density
CHO	Carbohydrate
CI	Confidence interval
CT	Computed tomography
DXA	Dual-energy X-ray absorptiometry
EC	Extracellular
eIF2α	Eukaryotic translation initiation factor 2 alpha
EWGSOP2	European Working Group on Sarcopenia in Older People 2
FFM	Fat-free mass
FOXO3a	Forkhead box O3a
H_2_O	Water
KB	Ketone bodies
KD	Ketogenic diet
LBM	Lean body mass
LEKD	Low-energy ketogenic diet
LST	Lean soft tissue
MASLD	Metabolic dysfunction-associated steatotic liver disease
MEDLINE	Medical Literature Analysis and Retrieval System Online
MF-BIA	Multifrequency bioelectrical impedance analysis
MRI	Magnetic resonance imaging
mRNA	Messenger ribonucleic acid
mTOR	Mechanistic target of rapamycin
mTORC1	Mechanistic target of rapamycin complex 1
NADH	Reduced nicotinamide adenine dinucleotide
PRISMA	Preferred Reporting Items for Systematic Reviews and Meta-Analyses
RCT	Randomised controlled trial
S6K	Ribosomal protein S6 kinase
S6K1	Ribosomal protein S6 kinase 1
SANRA	Scale for the Assessment of Narrative Review Articles
SGLT2	Sodium–glucose cotransporter 2
SMD	Standardised mean difference
T2D	Type 2 diabetes
VLCKD	Very-low-carbohydrate ketogenic diet
VLEKD	Very-low-energy ketogenic diet
VLEKT	Very-low-energy ketogenic therapy
WMD	Weighted mean difference
