# Supplementary material for: Sparing Muscle While Losing Fat: A Narrative Review of Ketogenic Diets and Lean Mass Preservation During Weight Loss
Source: Nutrients. 2026 Sep 7;18(17):2933. doi: 10.3390/nu18172933 (PMC13566994; doi:10.3390/nu18172933)
Supplement: Supplementary file 1 [file nutrients-18-02933-s001.zip › nutrients-4508867-supplementary.pdf]

Supplementary Table S1. Search documentation.

Search	Terms
**Search 1**<br>**Clinical ketogenic interventions and lean/muscle outcomes (325 records)**	("Diet, Ketogenic"[Mesh] OR ketogenic diet*[tiab] OR ketogenic therap*[tiab] OR "very low calorie ketogenic"[tiab] OR "very-low-calorie ketogenic"[tiab] OR "very low energy ketogenic"[tiab] OR "very-low-energy ketogenic"[tiab] OR "very low carbohydrate ketogenic"[tiab] OR "very-low-carbohydrate ketogenic"[tiab] OR VLCKD[tiab] OR VLEKD[tiab] OR VLEKT[tiab] OR "nutritional ketosis"[tiab]) AND ("Body Composition"[Mesh] OR "Muscle, Skeletal"[Mesh] OR "body composition"[tiab] OR "fat-free mass"[tiab] OR "fat free mass"[tiab] OR "lean body mass"[tiab] OR "lean mass"[tiab] OR "lean soft tissue"[tiab] OR "skeletal muscle"[tiab] OR "muscle mass"[tiab] OR strength[tiab] OR "physical function"[tiab] OR "physical performance"[tiab] OR sarcopen*[tiab] OR "muscle protein synthesis"[tiab] OR "protein turnover"[tiab] OR "muscle protein breakdown"[tiab] OR proteolysis[tiab] OR "nitrogen balance"[tiab]) AND ("Weight Loss"[Mesh] OR "Obesity"[Mesh] OR weight loss[tiab] OR weight reduction[tiab] OR obes*[tiab] OR overweight[tiab] OR energy restrict*[tiab] OR caloric restrict*[tiab] OR calorie restrict*[tiab]) AND ("1900/01/01"[Date - Publication] : "2026/08/14"[Date - Publication])
**Search 2**<br>**Ketone bodies and muscle protein turnover/signalling (192 records)**	("Ketone Bodies"[Mesh] OR ketone bod*[tiab] OR beta-hydroxybutyrate[tiab] OR beta-hydroxybutyric acid[tiab] OR 3-hydroxybutyrate[tiab] OR 3-hydroxybutyric acid[tiab] OR BHB[tiab] OR acetoacetate[tiab] OR acetoacetic acid[tiab] OR ketone ester*[tiab] OR ketone monoester*[tiab] OR ketone diester*[tiab] OR exogenous ketone*[tiab]) AND ("Muscle, Skeletal"[Mesh] OR skeletal muscle[tiab] OR muscle[tiab] OR myofibrillar[tiab]) AND (muscle protein[tiab] OR myofibrillar protein[tiab] OR protein synthesis[tiab] OR protein turnover[tiab] OR protein breakdown[tiab] OR proteolysis[tiab] OR leucine oxidation[tiab] OR leucine kinetic*[tiab] OR nitrogen balance[tiab] OR mTOR[tiab] OR mTORC1[tiab] OR FOXO3a[tiab] OR muscle atrophy[tiab] OR muscle wasting[tiab]) AND ("1900/01/01"[Date - Publication] : "2026/08/14"[Date - Publication])
**Search 3**<br>**Simplified AMPK-focused mechanism search (19 records)**	("Ketone Bodies"[Mesh] OR beta-hydroxybutyrate[tiab] OR BHB[tiab] OR acetoacetate[tiab]) AND ("Muscle, Skeletal"[Mesh] OR "skeletal muscle"[tiab]) AND (AMPK[tiab] OR "AMP-activated protein kinase"[tiab]) AND ("1900/01/01"[Date - Publication] : "2026/08/14"[Date - Publication])
**Search 4**	

**Measurement sensitivity to glycogen/hydration (53 records)**	((glycogen[Title] OR hydration[Title] OR "body water"[Title] OR "fluid shift"[Title]) AND ("dual-energy x-ray absorptiometry"[tiab] OR "dual energy x ray absorptiometry"[tiab] OR DXA[tiab] OR DEXA[tiab]) AND (body composition[tiab] OR lean mass[tiab] OR lean tissue[tiab] OR fat-free mass[tiab] OR fat free mass[tiab])) AND ("1900/01/01"[Date - Publication] : "2026/08/14"[Date - Publication])
**Search 5**<br>**Ketogenic/exogenous ketone and incretin combination (12 records)**	("Diet, Ketogenic"[Mesh] OR ketogenic diet*[tiab] OR nutritional ketosis[tiab] OR very-low-calorie ketogenic[tiab] OR very low calorie ketogenic[tiab] OR VLEKD[tiab] OR VLCKD[tiab] OR ketone ester*[tiab] OR ketone monoester*[tiab] OR exogenous ketone*[tiab] OR beta-hydroxybutyrate[tiab]) AND (GLP-1[tiab] OR GLP-1RA[tiab] OR glucagon-like peptide-1[tiab] OR incretin*[tiab] OR semaglutide[tiab] OR liraglutide[tiab] OR tirzepatide[tiab]) AND (body composition[tiab] OR fat-free mass[tiab] OR lean mass[tiab] OR skeletal muscle[tiab] OR muscle mass[tiab] OR strength[tiab]) AND ("1900/01/01"[Date - Publication] : "2026/08/14"[Date - Publication])

Database/interface: MEDLINE via PubMed.

Search date: 14 August 2026.

Coverage: database inception to 14 August 2026.

Core searches yielded 325 and 192 records. The initial long-form AMPK search returned zero records with a PubMed query-processing warning. The simplified Search 3 was then used.

Supplementary measurement and incretin searches yielded 53 and 12 records.

Forward citation searches yielded 144 unique records.

Supplementary Table S2. Study eligibility and prioritization.

Evidence group	Eligibility	Outcomes	Priority/interpretation
Core clinical	Adult ketogenic dietary intervention adequately described by carbohydrate/macronutrients, ketonaemia, or recognized VLEKD protocol	Body composition, anatomical muscle, strength/performance	Controlled trials and systematic reviews; retain original measurement terminology
Human mechanistic	Tracer, biopsy, or local/systemic ketone exposure	Protein synthesis/breakdown, nitrogen balance, signalling	Prioritize controlled human experiments; do not extrapolate exogenous ketones to dietary ketosis
Preclinical	Mechanistic question with absent/limited human evidence	Atrophy, signalling, phenotype hypotheses	Label explicitly as preclinical and hypothesis-generating
Contextual	Non-ketogenic low-carbohydrate, protein, exercise, measurement, pain, incretin, or safety evidence	Interpretation or implementation	Not primary evidence for a ketosis-specific effect
Excluded as primary evidence	Case reports; unsupported opinion; inadequately described exposure; no relevant outcome	—	May be mentioned only as clearly labelled context if uniquely informative
